# The Feasibility of a Co‐Designed Online Healthy Living Intervention for Cancer Survivors: Valuable Content, But Limited Engagement

**DOI:** 10.1002/pon.70486

**Published:** 2026-05-08

**Authors:** Morgan Leske, Bogda Koczwara, Jason Blunt, Camille E. Short, Anthony Daly, Julia N. Morris, Jon Degner, Lisa Beatty

**Affiliations:** ^1^ College of Human Sciences and Culture Flinders University Adelaide South Australia Australia; ^2^ Australian Research Centre for Cancer Survivorship University of New South Wales Sydney New South Wales Australia; ^3^ Cancer Council SA Eastwood South Australia Australia; ^4^ Faculty of Medicine, Dentistry and Health Sciences Melbourne Centre for Behaviour Change Melbourne School of Psychological Sciences and Melbourne School of Health Sciences The University of Melbourne Melbourne Victoria Australia; ^5^ Cancer Voices South Australia Kensington Park South Australia Australia

**Keywords:** cancer survivorship, digital health, feasibility, healthy lifestyle

## Abstract

**Introduction:**

Healthy Living after Cancer (HLaC) was an evidence‐based, telephone‐delivered lifestyle program for survivors. HLaC *Online* was co‐designed with cancer survivors, healthcare professionals, and cancer support representatives to enhance sustainability and reach through a self‐directed digital format, while incorporating both physical health and mental health content. This study examined HLaC *Online'*s feasibility.

**Methods:**

Eleven Australian post‐treatment cancer survivors participated in a 12‐week, single‐arm pilot with mixed‐methods evaluation. Feasibility indicators included uptake, usage, attrition, usability, satisfaction, and indicators of preliminary efficacy.

**Results:**

Of 318 website visitors, 11 enrolled, with nearly half never logging in. Module usage was limited (*M* = 3.3 of 9 modules) and only 5 completed follow‐up. Perceived usability of HLaC *Online* was varied (rated F− B+). Qualitative feedback highlighted the relevance of psychosocial modules, but engagement was hindered by technology barriers, cancer‐related symptoms, and lack of guidance. Participants suggested human support for orientation, accountability, and motivation.

**Conclusions:**

While HLaC *Online* was valued for its co‐designed mental health focus, feasibility was limited without guidance. Findings raise questions about which survivors can benefit from self‐directed digital programs versus those requiring human support. Future work should explore referral pathways, scalable human support models, and tailoring by need.

## 1 Background

Post‐treatment healthy lifestyle interventions supporting physical activity, nutrition, and/or weight management can reduce the risk of treatment‐related side effects, cancer recurrence, and mortality, while improving physical and emotional well‐being [1]. However, such interventions are not routinely implemented following cancer treatment. This gap is pronounced for those living in rural and remote areas who have reduced access to specialised healthcare. To address accessibility barriers, a telephone‐delivered healthy lifestyle intervention—Healthy Living after Cancer (HLaC)—was developed [2]. While HLaC yielded significant clinical benefits in improving physical activity, fruit and vegetable intake, and Quality of Life (QoL), the reliance on clinician‐delivered calls made the program too resource intensive and costly to be delivered beyond the funded trial [2].

Digital health modalities offer a potential solution by enabling health services to be self‐guided, continuously accessed regardless of time or location, and widely disseminated with minimal additional costs once developed [3]. Online platforms are of particular interest as they avoid the need for additional downloads or devices [3]. Therefore, to address sustainability barriers, the telephone‐delivered HLaC program was adapted into an online format (Healthy Living after Cancer Online; HLaC *Online*) using a co‐design approach, whereby post‐treatment cancer survivors, healthcare professionals, and cancer support representatives were involved at each stage of the intervention development and evaluation [4, 5].

Throughout the co‐design of HLaC *Online*, stakeholders highlighted the enduring psychosocial impact of a cancer diagnosis, including psychological distress and concerns about recurrence [4, 5]. Accordingly, they defined ‘healthy living’ as extending beyond physical activity, nutrition, and weight management, and encompassing mental health and adjustment following treatment. Psychosocial variables are often measured as outcomes of healthy living interventions, but few studies have explored these as intervention targets [6]. As a result, HLaC *Online* was designed as a multicomponent intervention which addresses the physical and psychosocial domains of a healthy lifestyle after cancer treatment [5]. This study evaluates the feasibility of the HLaC *Online* prototype.

## 2 Methods

### 2.1 Intervention

HLaC *Online* is a 12‐week online intervention supporting post‐treatment cancer survivors to achieve their healthy living goals (www.healthylivingaftercancer.org). It includes nine immediately accessible, self‐guided modules targeting goal setting, finding the new normal after cancer treatment, physical activity, healthy eating, mental health, fatigue management, maintaining a healthy weight, peer support, and adherence and maintenance. Based on Social Cognitive Theory [7], each module included an introduction video, cancer survivor story videos, psychoeducation, and interactive worksheets to develop evidence‐based behaviour change skills (e.g., goal setting, self‐monitoring, overcoming barriers, stimulus control, identifying social supports, and self‐reward; details in Supporting Information S1). The website was designed with a responsive format and could be accessed via a web browser on a computer, tablet, or phone.

### 2.2 Feasibility and Usability Evaluation

#### 2.2.1 Design

This study utilised a mixed methods design, including a single arm, pre‐post trial and a qualitative interview.

#### 2.2.2 Participants

Eligible participants were Australian adults (≥ 18 years), diagnosed with non‐metastatic cancer, curatively treated within the last 5 years, and had completed primary treatment. Those receiving hormonal treatment were still eligible. Exclusion criteria included contraindications for unsupervised physical activity (e.g., active heart disease), cognitive impairment, or limited English.

#### 2.2.3 Procedure

This study was approved by the Cancer Council Victoria Human Research Ethics Committee (HREC 2106) and registered with the Australian New Zealand Clinical Trials Registry (ACTRN12622001111763). Participants were recruited via social media advertisements and flyers disseminated by Cancer Council South Australia and Breast Cancer Network Australia. Participants completed self‐reported questionnaires via Qualtrics at baseline and post‐intervention (12 weeks). Access to the HLaC *Online* website was granted within two business days of baseline completion. At post‐intervention, participants were invited to a semi‐structured telephone interview.

#### 2.2.4 Measures

##### 2.2.4.1 Sociodemographics and Clinical History

Table 1 outlines the sociodemographic and clinical data collected.

**TABLE 1:** Participant sociodemographic and clinical characteristics (*N* = 11).

Characteristic	*M* (SD)
Age (years)	57.86 (9.84)
Body Mass Index	27.88 (5.95)
Age at diagnosis (years)	55.73 (9.25)
Time since diagnosis (years)	1.56 (0.99)

Characteristic	***n* (%)**
Gender	
Female	9 (81.82)
Male	2 (18.28)
Relationship status	
Married	7 (63.67)
Divorced	2 (18.18)
Widowed	2 (18.18)
Educational achievement	
Secondary school	2 (18.18)
TAFE	3 (27.27)
Tertiary	5 (45.45)
Employment status	
Employed	6 (54.55)
Retired	3 (27.27)
Unable to work	2 (18.18)
Country of birth	
Australia	8 (72.73)
South Africa	2 (18.18)
England	1 (9.09)
Geographical remoteness	
Urban	7 (63.67)
Regional/rural	4 (36.36)
Socio‐economic status (SES)^a^	
1st Quintile (most disadvantaged)	1 (9.09)
2nd Quintile	2 (18.18)
3rd Quintile	1 (9.09)
4th Quintile	5 (45.55)
5th Quintile (most advantaged)	2 (18.18)
Country of birth	
Australia	8 (72.72)
South Africa	2 (18.18)
England	1 (9.09)
Cancer type	
Breast	7 (63.67)
Other^c^	4 (36.36)
Completed treatment	
Yes	7 (63.67)
No/unsure^d^	4 (36.36)
Treatment received^b^	
Surgery	4 (36.36)
Chemotherapy	5 (45.55)
Radiotherapy	7 (63.67)
Immunotherapy	1 (9.09)
Hormonal therapy	4 (36.37)

^a^ An area level indication of socio‐economic status measured using the Australian Bureau of Statistics Index of Relative Socio‐economic Advantage and Disadvantage derived from participants' postal codes.

^b^ Multiple responses allowed.

^c^ Colorectal (*n* = 1), head and neck (*n* = 1), lymphoma (*n* = 1), and prostate (*n* = 1).

^d^ All participants which selected ‘No’ or ‘Unsure’ for currently undertaking treatment indicated they were currently on hormonal treatment.

##### 2.2.4.2 Primary Outcome: Feasibility

HLaC *Online* feasibility was determined by (1): intervention uptake (i.e., proportion of unique visitors who register); (2) usage of the intervention (i.e., number of logins, pages viewed, and modules accessed); (3) attrition at post‐intervention survey; (4) perceived usability of intervention (System Usability Scale, with scores graded using the Sauro‐Lewis curved grading system [8]), and (5) satisfaction (explored qualitatively).

##### 2.2.4.3 Secondary Outcome: Preliminary Efficacy

Preliminary efficacy outcomes were QoL (Functional Assessment of Cancer Therapy—General [9]), moderate and vigorous physical activity (MVPA; Active Australia Survey [10]), average daily sitting time (International Physical Activity Questionnaire—sitting items [11]), diet quality (Fat and Fibre Behavioural Questionnaire [12]), fatigue (Functional Assessment of Chronic Illness Therapy—Fatigue Scale [13]), distress (Depression, Anxiety, and Stress Scale [14]), fear of cancer recurrence (4‐item Concerns About Recurrence Questionnaire [15]), and cancer‐related symptom severity and interference (MD Anderson Symptom Inventory [16]). Due to a coding error, the Fat and Fibre Behavioural Questionnaire could not be used. Two open‐ended items measuring fruit and vegetable consumption, provided a proxy for diet quality.

### 2.3 Statistical Analysis

Quantitative analysis was conducted using SPSS 28. Descriptive statistics summarised participant's sociodemographic, clinical characteristics and feasibility outcomes. Chi‐squared analyses and independent samples *t*‐tests compared baseline differences between post‐intervention questionnaire completers and non‐completers. Pre‐post changes in preliminary efficacy outcomes were examined using matched paired‐samples *t*‐tests with estimates of Cohen's *d*, corrected for correlated observations. Due to the small sample size, results were interpreted with respect to the magnitude and direction of effect sizes, rather than inferential statistics, and interpreted as small (0.2), medium (0.5), or large (0.8) effects. Negative effect sizes indicate higher mean scores at post‐intervention.

Interviews were transcribed verbatim. Deductive thematic analysis was used to understand participants subjective experiences and summarise their recommendations for intervention improvement. *A priori* themes based on Bowen and colleagues' [17] feasibility framework included acceptability (i.e., suitability and satisfaction with HLaC *Online*), demand (i.e., usage or intended usage), and practicality (i.e., resources, time, and commitment required for participation). To mitigate bias, themes created by one investigator (M.L.) were refined with another author (L.B.) experienced in qualitative analysis and evaluating online psycho‐oncology interventions.

## 3 Results

### 3.1 Feasibility Outcomes

#### 3.1.1 Uptake

Of the 318 unique visitors to the website, 16 (5%) registered for HLaC *Online* and 11 (68.8%) completed the baseline questionnaire and were eligible to participate. Baseline sociodemographic and clinical characteristics of enrolled participants are summarised in Table 1. Two were ineligible as they were more than 5 years post‐diagnosis. Following baseline, five participants (45%) never logged into HLaC *Online*, and the automated email reminder process was not triggered.

#### 3.1.2 Attrition

Six (54.5%) participants did not complete the post‐intervention questionnaire. The average MVPA of participants who did not complete the post‐intervention questionnaire (*M* = 260.0 minutes, SD = 193.18) was lower than participants who completed the questionnaire (*M* = 406.0 minutes, SD = 457.69, *d* = 0.43). No other differences in sociodemographic, clinical or psychosocial characteristics were observed.

#### 3.1.3 Usage

Among the six participants who logged into the program, module engagement was low, with most accessing one to four modules (*M* = 3.33, SD = 3.01). One participant accessed all nine modules. The most popular modules were *Finding the New Normal*, *My Goals* and *Physical Activity* modules, each accessed by three participants. On average, participants logged in 4.3 times (range = 0–29) and accessed 8.46 pages (range = 0–57).

#### 3.1.4 Website Usability

Three participants rated usability above average (C− B+). One participant rated program usability as an F.

### 3.2 Indicative Effect Sizes

At post‐intervention, effect sizes indicated an increase in physical activity and vegetable intake, and a decrease in psychological distress (Table 2). There were moderate decreases in Global QoL and Fatigue, with substantial variability across participants over time. No other outcomes demonstrated a change over time.

**TABLE 2:** Pre‐post effect sizes for indicators of efficacy outcomes (*N* = 5).

Scale	Baseline *M* (SD)	Post‐intervention *M* (SD)	Cohen's *d*	95% CI	
				Lower	Upper
QoL	83.20 (9.73)	78.67 (5.73)	0.45	−0.40	1.30
Fatigue	37.00 (10.65)	34.00 (9.70)	0.91	−0.20	1.93
Physical activity (minutes)	406.00 (457.69)	618.00 (545.64)	−0.42^a^	−2.20	1.36
Average daily sitting time (minutes)	298.29 (175.60)	267.43 (149.50)	0.30^a^	−0.62	1.18
Diet quality					
Vegetable servings/day	2.80 (0.84)	3.20 (1.34)	−0.45^a^	−1.35	0.50
Fruit servings/day	3.00 (0.71)	3.20 (0.84)	0.00	−0.88	0.88
Distress	23.60 (20.90)	8.40 (5.73)	0.67^a^	−0.49	1.84
Fear of cancer recurrence	12.20 (9.76)	11.40 (5.55)	0.06	−0.46	0.59
Symptom severity	3.96 (1.92)	3.78 (1.27)	0.09	−0.59	0.76
Symptom interference	3.20 (2.66)	2.99 (1.32)	0.03	−0.22	0.29

*Note:* Negative effect sizes indicate a higher score at post‐treatment.

^a^ Indicates that the effect size direction shows improvements at post intervention.

### 3.3 Qualitative Findings

Three participants completed a telephone qualitative interview and one responded to qualitative questions via email. Mean age (*M* = 53.69, SD = 2.52) and time since diagnosis (*M* = 1.27, SD = 0.70) was reflective of the overall sample. Three participants had breast cancer, and one had lymphoma. Three participants were currently employed, and one was unable to work.

### 3.4 Acceptability

One participant reported satisfaction with the program, whereas the remaining three participants reported being neither satisfied nor unsatisfied, citing limited program use. Acceptability was influenced by the [1] program's self‐directed format and accessibility; and [2] whether HLaC *Online* met their information needs.

Two participants appreciated the flexibility for choosing modules but sometimes felt unsure about what to do next due to the number of available modules and missed helpful content, such as using the thought record during a hospital check‐up: ‘I should have had a look at this and used this tool. It probably would have helped’ (Breast Cancer Survivor 1). Another participant suggested a mobile application would improve intervention accessibility.

Participants had mixed views on whether HLaC *Online* met their informational needs. Two found the *Physical Activity* and *Healthy Eating* modules helpful for behaviour change: ‘it's those two things [that] have probably had the biggest impact on me because it's changed what I'm eating, but it's also made me go and join an exercise programme because I know I'm not walking enough now’ (Lymphoma Survivor). Another participant stated that the content reinforced their existing knowledge. They indicated a preference for more scientific explanations though acknowledged this may not suit all users. Additional topics requested included returning to work, high‐intensity exercise, and resources for family and friends.

### 3.5 Demand

Demand for HLaC *Online* was determined by the participant's self‐assessment that their needs were already met by other applications or services. Two participants using other health applications (e.g., FitBit) or professional supports reported less engagement with the program. All participants found the Finding the New Normal module and the cancer survivor story videos were the most helpful components.

### 3.6 Practicality

The practicality of HLaC *Online* was impacted by competing priorities, such as work commitments and cancer‐related symptoms (e.g., fatigue and cognitive impairment): ‘I was firmly on track to look at all the resources and then I just had a lack of energy. Like I felt I had to reserve the energy and motivation that I have for my daily requirements and that was just an extra thing that I just didn't have the time or energy for’ (Breast Cancer Survivor 3). Furthermore, website glitches reduced willingness to participate. Email reminders were viewed as helpful, though one participant suggested SMS reminders for better visibility. Another suggestion was offering human support for program navigation and motivation, particularly for those experiencing cancer‐related symptoms.

## 4 Discussion

This study aimed to evaluate the feasibility of the HLaC *Online* prototype. It builds upon previous co‐design stages [4, 5] by understanding how end‐users interact with HLaC *Online* over the proposed intervention period. The co‐designed content addressing the psychosocial challenges of post‐treatment survivorship was well received. However, low engagement was a key barrier to HLaC *Online's* feasibility. Low engagement was primarily driven by barriers to initial access (i.e., registration and first login), rather than disengagement during the intervention period, as nearly half of enrolled participants did not login to the program. Similar online healthy lifestyle interventions have demonstrated a considerably higher engagement rate, with up to 90% of participants logging into the program at least once [18]. Nevertheless, among participants who successfully logged into HLaC *Online*, module usage was comparable to previous studies that have observed positive outcomes. This includes interventions which provided personalised module recommendations based on participant's baseline data [19]. These findings suggest that selective engagement with a subset of relevant content may be sufficient to support behaviour change in multicomponent digital interventions, at least for some survivors.

### 4.1 Clinical Implications

While self‐directed online interventions can be effective [20], some individuals may require additional support to engage with them, including assistance during initial access and ongoing support throughout the intervention period. One recommended solution is implementing human support alongside the online program to boost engagement through accountability, module selection, and tailored feedback. Low intensity guidance through two telephone calls has shown promise in boosting engagement and outcomes [21]. Next steps will explore options for scalable human support and determine for whom and under what conditions are self‐directed models appropriate to inform a stratified care approach that triages additional support according to individual need.

### 4.2 Limitations

Limitations include the small, homogenous sample and absence of feedback from non‐engagers of HLaC *Online*, which limited the ability to confirm whether reasons for low engagement also contributed to *non‐engagement*. Future studies should consider strategies to capture feedback from both engagers and non‐engagers during the intervention period to clarify early barriers to engagement.

### 4.3 Conclusion

While the co‐designed approach produced valuable *content* for HLaC *Online*, this approach has not yet addressed how to *deliver* this intervention in a user‐friendly, engaging, and economically sustainable way. Future work is needed to determine scalable human support and explore options for tailoring and stratified care.

## Author Contributions

All authors contributed to the study conception, design, and interpretation. Data collection and analysis were performed by Morgan Leske. Morgan Leske drafted the main manuscript text, and all authors provided feedback on previous drafts. All authors read and approved the final manuscript.

## Funding

This project was funding by the Flinders University Innovation Partnership Seed Grant and Cancer Council SA.

## Ethics Statement

This study was approved by the Cancer Council Victoria Human Research Ethics Committee (HREC 2106) and received cross‐institutional approval from Flinders University Human Research Ethics Committee (4982).

## Consent

Informed consent was obtained from all individual participants included in the study.

## Conflicts of Interest

The authors declare no conflicts of interest.

## Supporting information


Supporting Information S1

## Data Availability

The data that support the findings of this study are available from the corresponding author upon reasonable request.
