# Supplementary material for: The Feasibility of a Co‐Designed Online Healthy Living Intervention for Cancer Survivors: Valuable Content, But Limited Engagement
Source: Psychooncology. 2026 May 8;35:e70486. doi: 10.1002/pon.70486 (PMC13154715; doi:10.1002/pon.70486)
Supplement: Supplementary file 1 — Supporting Information S1 [file PON-35-e70486-s001.docx]

**Supporting Information 1**

**Title: Feasibility of a co-designed online healthy living program for cancer survivors: a pilot mixed methods trial**

Figure 1
*HLaC Online home screen*


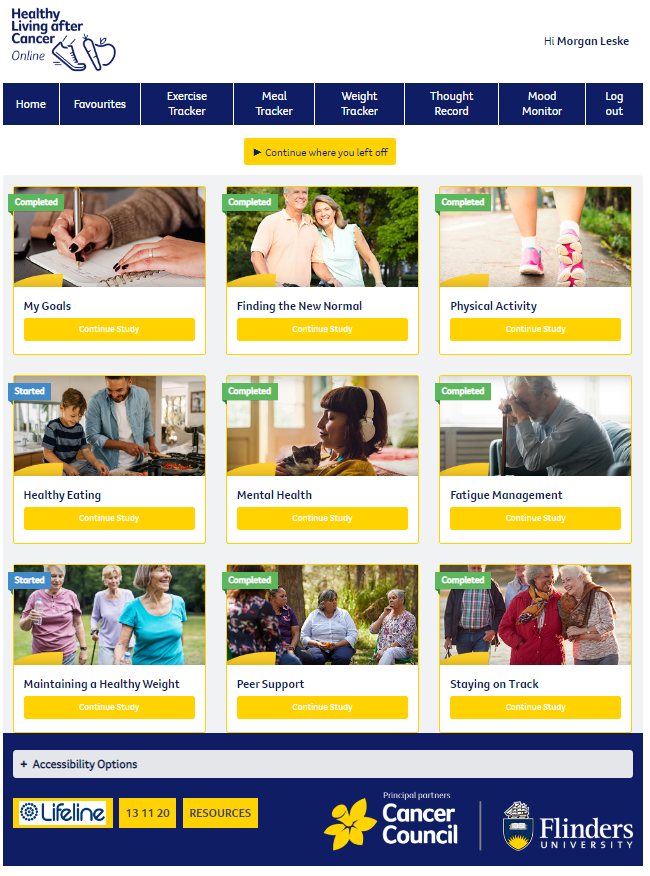


**Table 1.** Module content from Healthy Living after Cancer Online

| **Module** | **Description** | | **Activities** | |  |
| --- | --- | --- | --- | --- | --- |
| *My Goals* | Covers techniques based on motivational interviewing and goal setting to support participants in the identification of health behaviours to change and the development of their healthy living goals. To ensure that the participants goals are clear and reachable, this module utilised SMART goals, that is, goals that are:   - **S**pecific (what are they trying to achieve?) - **M**easurable (how will they measure their progress?) - **A**ctionable (what do they need to achieve this goal?) - **R**ealistic (are they able to achieve this goal?) - **T**imely (when will they achieve this goal?) | | - What are your aims for participating the Healthy Living after Cancer Online? - List positives and negatives of current health behaviours and changing behaviours. - Why is change important to me? - Create your own SMART goals. - Develop an action plan (i.e., three actionable steps to achieve SMART goal). | |  |
| **Table 1 *continued*** | | | | |  |
| **Module** | **Description** | | **Activities** | |  |
| *Finding the new normal* | Provides information about the common experiences of ending treatment, including different myths, e.g., ‘I should feel well’, and ‘I should not need support’ (Cancer Council 4), treatment related side effects (e.g., fatigue, pain, loss of self-esteem), and returning to work. | | - Video series of cancer survivors sharing their experience of completing treatment. - Links to a return-to-work plan and information about how workplaces can support returning to work. | |  |
| *Physical activity* | In line with the Clinical Oncology Society of Australia’s (2018) position statement on physical activity, HLaC *Online* recommends meeting or exceeding 30 minutes per day of moderate to vigorous physical activity. Participants are encouraged to engaged in both planned and incidental physical activity (e.g., taking the stairs instead of the lift). This module covers: (a) the benefits of exercise, (b) different types of physical activity (aerobic exercise, strength training, flexibility), (c) how to build a strength training session (including push muscles such as chest, shoulders, and triceps; and pull muscles such as back and biceps, a lower body exercise (i.e., | | - Video of cancer survivors sharing their experience engaging in physical activity after treatment. - What benefits would you get from being more active? - What kind of physical activity do you enjoy?   Instructional videos of weighted or body weight exercises.  Accessing support for exercise (e.g., exercise physiologist or personal trainer) | |  |
| **Table 1 *continued*** | | | | |  |
| **Module** | **Description** | | **Activities** | |  |
|  | quads, hamstrings, and calves), a core and balance exercise, and a cool down). To ensure that the exercises are accessible to all participants, this module primarily focuses on exercises that do not require equipment. | |  | |  |
| *Healthy eating* | The major dietary aims of HLaC *Online* are to:  (1) increase intake of fruit, vegetables, and wholegrains;  (2) reduce intake of added sugars and saturated fats;  (3) limit portion sizes and making healthy food choices.  The module provides information on food guidelines, making food swaps, and portion control (i.e., lowering the size or number of serves). | | - Video of cancer survivors sharing their experience on making healthier food choices. - How to read a food label. - Which food swaps will you try? - What eating away from home strategies will you try? - Accessing support from a dietitian | |  |
| **Table 1 *continued*** | | | |  |  |
| **Module** | **Description** | | **Activities** |  |  |
| *Mental Health* | Focuses on the emotional experience of finishing cancer treatment. Specifically, this module highlights the wide range of emotions participants experience, including the re-emergence of life stressors that have been deferred, coping with treatment-related side effects or body changes (e.g., scaring from surgery), and common mental health concerns (depression, anxiety, fear of cancer recurrence or cancer progression, and distress about body image). This module also provides strategies for managing distress based on cognitive behaviour therapy and mindfulness-based stress reduction. | | - Thought challenge record - How to practice mindfulness - Audio recordings of guided meditations - Accessing support from a counsellor or psychologist | |  |
| *Fatigue management* | Provides information about the common symptoms of fatigue, possible causes, and strategies for daily and long-term fatigue management. | | - Using the ‘Three P’s’ for daily energy management: plan, prioritise, and pace | |  |
| **Table 1 *continued*** | | | |  |  |
| **Module** | **Description** | | **Activities** |  |  |
| *Maintaining a healthy weight* | Assists the participant in identifying whether they are in a healthy weight range and provides two subsequent sections on weight loss or weight gain. The section on weight loss provides information about modest weight loss (i.e., 5 – 10% of initial body weight) by reducing energy intake by 2000kj per day. Strategies to reduce energy intake included portion control and lowering energy density (i.e., by replacing high energy dense foods, such as high fat or sugar foods, with low energy dense foods, such as fruit and vegetables). The weight gain section covers strategies that help with loss of appetite (e.g., establishing a regular eating pattern and small frequent meals), food swaps to increase energy intake, and food type nutritional supplements. | | - Finding out if you are a healthy weight using a BMI calculator and measuring your waist. | |  |
| **Table 1 *continued*** | | | | | |
| **Module** | **Description** | **Activities** | | | |
| *Peer support* | This module provides information about different peer support avenues, including support groups, volunteer opportunities to support others with cancer (e.g., Cancer Voices), Cancer Connect (a telephone service offered by Cancer Council to connect people with a trained volunteer with a similar cancer diagnosis), the Cancer Council Online Community and Facebook groups. | | - Links to each state Cancer Council support group pages - Link to Cancer Voices Australia - Link to the Cancer Council Online Community | |  |
| *Staying on track* | This module aims to support participants in maintaining their healthy lifestyle changes. This module includes information about habit formation, planning ahead for events where you may stop engaging in health behaviours (e.g., holidays), what to do if you experience a ‘slip,’ getting support from others, and celebrating success. | | - Where is your best source of support for healthy living? - How will you celebrate your success? | |  |
